# Design and evaluation protocol of "FATaintPHAT", a computer-tailored intervention to prevent excessive weight gain in adolescents

**DOI:** 10.1186/1471-2458-7-324

**Published:** 2007-11-12

**Authors:** Nicole PM Ezendam, Anke Oenema, Petra M van de Looij-Jansen, Johannes Brug

**Affiliations:** 1 Department of Public Health, Erasmus University Medical Center, Rotterdam, The Netherlands; 2 Municipal Public Health Service for Rotterdam area, Rotterdam, The Netherlands; 3 EMGO Institute, VU University Medical Center, Amsterdam, The Netherlands

## Abstract

**Background:**

Computer tailoring may be a promising technique for prevention of overweight in adolescents. However, very few well-developed, evidence-based computer-tailored interventions are available for this target group. We developed and evaluated a computer-tailored intervention for adolescents targeting energy balance-related behaviours: i.e. consumption of snacks, sugar-sweetened beverages, fruit, vegetables, and fibre, physical activity, and sedentary behaviours. This paper describes the planned development of a school-based computer-tailored intervention aimed at improving energy balance-related behaviours in order to prevent excessive weight gain in adolescents, and the protocol for evaluating this intervention.

**Methods/design:**

Intervention development: Informed by the Precaution Adoption Process Model and the Theory of Planned Behaviour, the computer-tailored intervention provided feedback on personal behaviour and suggestions on how to modify it. The intervention (VETisnietVET translated as 'FATaintPHAT') has been developed for use in the first year of secondary school during eight lessons.

Evaluation design: The intervention will be evaluated in a cluster-randomised trial including 20 schools with a 4-months and a 2-years follow-up. Outcome measures are BMI, waist circumference, energy balance-related behaviours, and potential determinants of these behaviours. Process measures are appreciation of and satisfaction with the program, exposure to the program's content, and implementation facilitators and barriers measured among students and teachers.

**Discussion:**

This project resulted in a theory and evidence-based intervention that can be implemented in a school setting. A large-scale randomised controlled trial with a short and long-term follow-up will provide sound statements about the effectiveness of this computer-tailored intervention in adolescents.

**Trial Registration:**

ISRCTN15743786

## Background

Increasing numbers of adolescents worldwide are overweight and obese [1]. In the Netherlands 22% of girls and 18% of boys are considered overweight or obese [2]. Obesity is associated with an increased risk of chronic diseases (e.g. cardiovascular disease, type 2 diabetes, and cancer), and some of these may already become manifest at an early age. Overweight and obese adolescents are likely to become overweight or obese adults [1,3,4]. Furthermore, treatment of overweight and obesity is generally disappointing [1,5]. To prevent adolescents from becoming overweight or obese, there is an urgent need for interventions aimed at preventing excessive weight gain in adolescents.

Overweight is the result of a long-term imbalance between energy intake (through the diet) and energy expenditure (mainly through physical activity). Interventions aimed at prevention of excessive weight gain should preferably target both sides of the energy balance. Various specific behaviours regarding diet and physical activity have been suggested to be related to the energy balance: i.e. the consumption of snacks, sugar-sweetened beverages, fruit, vegetables, and fibre, as well as physical activity, television viewing and computer use [6-10]. These behaviours should therefore be targeted in interventions.

Computer tailoring is a promising health communication technique to alter dietary and physical activity behaviours in adults [11]. The technique may also be suitable for modifying energy balance-related behaviours (EBRBs) in adolescents [12]. Computer tailoring is intended to reach one specific person by addressing characteristics that are unique to that person, related to a specific behaviour, and derived from an individual assessment [13]. The theoretical rationale behind computer tailoring is that non-personally relevant information is eliminated as much as possible. Therefore, people will pay more attention to the information, appreciate the information more, and find it more interesting. Theories of information processing (such as the Elaboration Likelihood Model), show that information that is attended to and thoughtfully considered is more likely to influence the person's awareness, knowledge, attitudes, beliefs, and behaviours [13]. Thus, computer tailoring might be a useful strategy to use for prevention of excessive weight gain in adolescents. However, very few studies have explored the effectiveness of computer-tailored interventions in adolescents.

Intervention development should follow a planned approach using theories and existing evidence [14,15]. The outcomes of this planned approach should be made available for researchers to support a profound interpretation of the study results, and to enhance the development of future interventions [11,14]. We developed a web-based computer-tailored intervention (CTI) named VETisnietVET (the closest English translation would be FATaintPHAT), which was used during first-year lessons at secondary schools and aimed at prevention of excessive weight gain. The school setting provides a natural learning environment for adolescents and ensures that all adolescents are exposed to the intervention. A planned, theory and evidence-based approach to intervention development was taken, and the intervention will be evaluated on the short and longer term. This paper describes the steps in the planned development of the intervention and the study design for evaluating the effectiveness of the intervention.

## Methods/design

### The intervention

#### Intervention objectives and behavioural goals

The objective of the intervention was to prevent excessive weight gain among adolescents aged 12 to 13 years. The increasing autonomy during adolescence might provide a window of opportunity to acquire healthy diet and physical activity patterns. Adolescents from diverse ethnic backgrounds (about half of the adolescents in the research area are from non-Dutch origin), school levels (from vocational to pre-university education) and socio-economic positions (from areas with various socio-economic characteristics) were the focus of the intervention. To achieve the intervention objective, the CTI aimed at improving the following EBRBs: consumption of sugar-sweetened beverages, snacks, fruit, vegetables, and fibre, as well as television/computer use, and physical activity [16-19]. For each behaviour cut-off points were set based on literature research, the recommendations of the National Nutrition Centre, and on the Dutch norms for physical activity [20] (Table 1).

**Table 1:** Recommendations and cut-off points used to provide feedback on the student's behaviour

**Behaviour**	**Cut-off points applied**
Consumption of sugar-sweetened beverages and fruit juice	Maximum of two glasses per day (approximately 400 ml)
Snack consumption	Maximum of three in-between-meal moments of eating, where the calories from relatively unhealthy snacks should not exceed the number of calories from relatively healthy snacks.
Fruit consumption	Minimum of two portions of fruit per day
Vegetable consumption	Minimum of 200 grams of vegetables per day (4 tablespoons)
Fibre consumption	Whole wheat bread (instead of white bread), breakfast with bread or cereal, rice/pasta/potato with dinner, four tablespoons of vegetables, and two pieces of fruit per day
Physical activity	At least one hour of moderate to vigorous intensity physical activity per day
Television and computer use	No more than two hours of television viewing and computer use combined per day

#### Theoretical background and determinants addressed

The theoretical model used as the basis of the intervention was a combination of the Precaution Adoption Process Model (PAPM) and the Theory of Planned Behaviour (TPB). These theories define important and changeable determinants to be addressed in the intervention. According to the PAPM, awareness of one's risk behaviour is an important determinant for the intention to change [21]. Lack of awareness is a determinant that is often an issue in complex health-related behaviour, such as dietary intake and physical activity [22,23]. Important determinants from the TPB that were targeted in the intervention are attitude, subjective norm, perceived behavioural control, and intention to change. In addition, other psychosocial constructs identified as being relevant in earlier studies were addressed, namely social support, skills, and planning.

#### General intervention framework

We implemented the Internet-delivered intervention in the school setting to reach all students (from different socio-economic and ethnic groups) and to present the intervention in a natural learning environment. The intervention website consisted of eight modules that could be used and completed in separate sessions. The homepage introduced the purpose and the importance of the FATaintPHAT program, and how the program could be used. The first module introduced the concept of weight, weight gain and related behaviours, and included an assessment of and feedback on an individual's BMI (Body Mass Index). Each of the other seven modules addressed one of the seven relevant behaviours. Every module consisted of an assessment questionnaire, individually-tailored feedback and advice, and an element to formulate an implementation intention. The feedback was presented on the computer screen immediately after the questionnaire had been completed and could be printed. Each subsequent module could be accessed after the previous one had been completed. The students could work through one session per lesson, and the whole program could be worked through in eight lessons. For each module the resulting feedback, advice, and formulated implementation intention could be read again in the module "Your advice".

**Figure 1:**
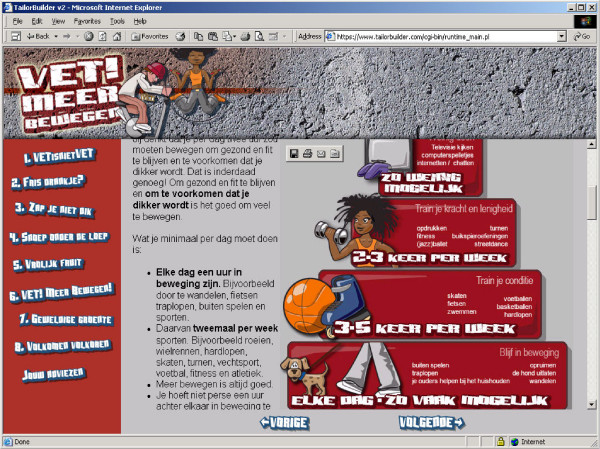
Screenshot of the FATaintPHAT intervention.

To increase the likelihood that the intervention would be adopted, we involved students and teachers in various parts of the development process. With 10 adolescents we discussed various possible titles for the intervention website; they preferred the name FATaintPHAT and understood the meaning. We also asked their opinions about different lay-outs for the website (designed especially for adolescents by a design bureau) to assess acceptability of the lay-outs, and whether there was a difference between boys and girls in level of acceptability. The lay-out as shown in Figure 1 emerged as the favourite, although it was appreciated more by the boys than by the girls. After extensive testing for accuracy by the researchers, nine experts in the field of nutrition, physical activity, and health education tested the website and commented on its content. Based on their feedback we modified the questions and feedback messages.

To prevent the intervention from inducing worrisome dieting behaviours in adolescents with a low BMI [24], underweight girls and boys received neutral information about healthy eating and physical activity, and were not advised to reduce their energy intake or to increase their physical activity level based on their diet and physical activity patterns. Being underweight was identified based on the BMI test that was part of the intervention and was defined as having a BMI below the cut-off points in the Dutch population as described previously [25].

#### Content of the computer tailoring program

The tailoring program consisted of assessment instruments, feedback libraries, tailoring algorithms to link up the outcomes of the assessment with the relevant messages in the feedback library, and a webpage to present the complete tailored communication [13,26]. To build the program we used the Tailorbuilder software (OSE, Sittard, The Netherlands [27]), which was specifically developed for production of Internet-delivered computer-tailored programs.

A screening instrument for each separate behaviour was developed that assessed the behaviour, perception of the behaviour, and the determinants related to that behaviour. Where possible we used parts of existing computer-tailored interventions. After completion of the questionnaire, feedback on a knowledge test and on personal behaviour in relation to the behaviour-specific recommendation was provided. Subsequently, feedback was provided on intention, attitudes, subjective norm, and perceived behavioural control. Students in the early stages of the PAPM received feedback to promote changes in attitudes, whereas for students in the later stages of the PAPM the focus was on perceived behavioural control. For improving perceived behavioural control, feedback on social support, skills, and planning was provided.

To promote changes in these behavioural determinants, a choice can be made from a variety of methods. A method is defined as a general technique or process to influence changes in the determinants of behaviours (and environmental conditions) [28]. An overview of the methods that were used is given in Table 2, as well as examples of feedback messages in which this method was applied.

**Table 2:** Overview and examples of the methods used in the feedback messages of the FATaintPHAT intervention

**Determinant**	**Methods**	**Example from FATaintPHAT**
**Knowledge**	• Provide factual information • Multiple-choice knowledge test	Do you know how many sugar cubes there are in a single glass of Coke? There are 4 of them! You have to jog for 15 minutes to burn up this sugar!
**Awareness of risk behaviour**	• Personal feedback • Normative feedback • Comparison with peers	Your answers show that you are watching television/using the computer for three hours a day. You think that this is not that much, but it is really too much!
**Intention to change**	• Re-evaluation of intention to change behaviour	Intending to exercise more? You say you haven't really thought about it. But maybe you've changed your mind, knowing that you exercise too little.
**Attitude towards behavioural change**	• Reinforce existing beliefs • Re-evaluation of beliefs • Present new arguments • Present counterarguments • Dismantle prejudices	Exercising is sweating. And you don't like that. Exercising makes you warm and to loose your warmth you need to sweat. That's very normal. Sweating means you are doing something good and nice! And is sweating really so bad... when you can take a nice shower afterwards?
**Perceived behavioural control**	• Provide practical tips and tricks • Discussion of difficult situations • Organizing environmental and social support	Choose to eat your fruit at fixed moments of the day, you won't forget it then, e.g.: - In between meals: you can eat fruit instead of candy or cake. - Breakfast: you can put fruit on your bread and with your yoghurt or milk. Think of bananas or strawberries.
**Planning**	• Formulate implementation intention	Example: *What? *Eat a piece of fruit instead of a candy bar *Where? *At school *When? *During the short break in the morning. Now make your own plan!

#### Pre-test of the intervention

A pre-final version of the intervention was pre-tested for usability, comprehension and acceptability among a small group of adolescents aged 12–14 years. Participants for the pre-test were recruited from two classes of two different schools (vocational and pre-university). Quantitative research was carried out among the vocational school students using questionnaires addressing the usability, appreciation, comprehension, and time needed to complete each module. Qualitative research was carried out using 'think aloud' sessions with 15 students of the pre-university class, to get detailed information about the usability and comprehension of the intervention. The results of the qualitative and quantitative pre-test showed that usability, acceptability and comprehensibility were acceptable. Minor adaptations to improve text comprehension (e.g. the difference between soft drinks and fruit juice), usability (e.g. a missing 'next' button), and tailoring algorithms were made. In addition, the pre-test demonstrated the time needed to complete each module.

### Evaluation design

#### Objectives and design

**Figure 2:**
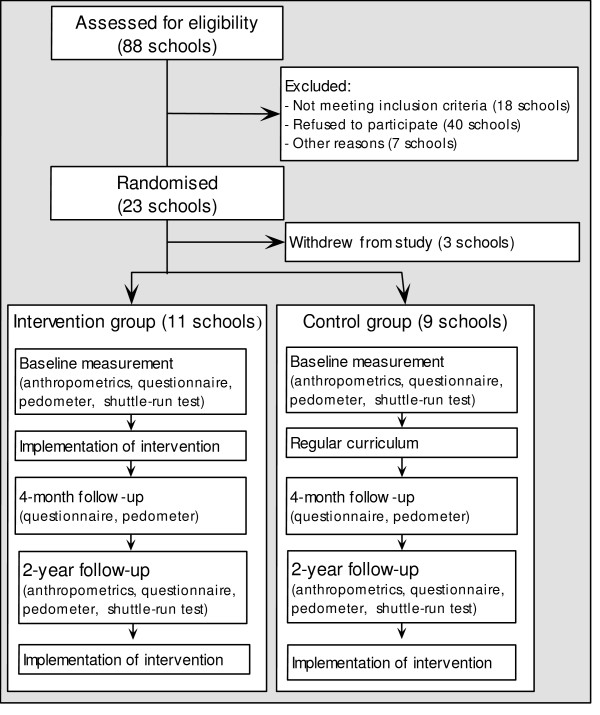
Flow chart of the recruitment and randomisation of schools.

The effectiveness of the intervention will be evaluated in a two-group (intervention and control) cluster-randomised trial with a 4-months and 2-years follow-up, among adolescents aged 12 – 13 years at the start of the study (Figure 2). The outcome measures are BMI and waist circumference (at 2-years follow-up), and the EBRBs and their determinants (at 4-months and at 2-years follow-up). Hypotheses to be tested are:

1. The intervention group has a lower BMI and waist circumference, compared to the control group at 2-years follow-up.

2. The intervention group has more favourable outcomes on the targeted behaviours, compared to the control group at 4-months and 2-years follow-up.

3. The intervention group is more aware of their risk behaviours and has more positive attitudes, perceived behavioural control and intentions toward changing their risk behaviours, compared to the control group at 4-months and 2-years follow-up.

4. Availability and accessibility of foods and physical activity opportunities in the home and school environment moderate the intervention effects.

In addition to testing the main hypotheses, secondary analyses will be performed to answer other relevant research questions.

The evaluation of effects will be accompanied by a process evaluation. Process level outcome measures are appreciation of and satisfaction with the intervention content, implementation rates (how well the intervention is implemented in school lessons) and exposure to the intervention content, and opinions of teachers about barriers and facilitators for intervention implementation.

The study was approved by the Medical Ethics Committee of the Erasmus MC.

#### Recruitment of schools and students

##### Recruitment of schools and classes

Recruitment of schools and logistics of the study was organized in collaboration with the municipal health organisations, who also sent the first invitational letter or email to the 88 schools in Rotterdam and surrounding municipalities in the Netherlands. The letter or email explained the purpose, design and logistics of the study and asked school directors to consider participation. Thereupon, a researcher telephoned all schools to provide additional information and asked for willingness to participate. If schools were interested to participate, eligibility was assessed by checking that the school did not meet the criteria for exclusion. The exclusion criteria concerned offering special sports education, having students who are not able to fill in a questionnaire, and having scheduled other interventions for weight control. Four classes of each school were randomly selected to participate, although in five schools the school representative selected participating classes non-randomly, because of time schedules and computer room availability.

To have sufficient power to detect a relevant difference of 1.0 kg (standard deviation 5 kg) in mean bodyweight between intervention and control group with power 0.80 and alpha 0.05, a minimum of 20 participating schools and four classes within each school was needed. This takes into account the cluster-randomised design, unreturned informed consent forms, refusal to participate, and expected drop-out during follow-up. Twenty-three schools were willing to participate in the study (Figure 2).

##### Randomisation procedure

Schools were randomly assigned to either the intervention or control group, using block randomisation. Block randomisation allows to start with the study while still including schools. Schools were stratified according to educational level, with vocational education in one stratum and higher than vocational education in the other stratum. Eleven schools were randomised in the intervention group (7 vocational; 4 higher than vocational) and twelve in the control group (7 vocational; 5 higher than vocational). Three schools withdrew from the study before the baseline measurement (2 vocational, 1 higher than vocational; all from the control group). Reason for withdrawal was experiencing the informed consent procedure as troublesome, and return of consent forms close to zero percent.

##### Recruitment of adolescents

During the school year, one school after the other started with the research activities. Per school, all the students in the selected classes were invited to participate. A researcher or teacher briefly explained the purpose and procedure of the study in the classroom. In addition, information letters providing details about the study and informed consent forms for the students and the parents were handed out. The adolescents were asked to give the information letter and the consent form to their parents and return the completed forms to the teacher. A total of 1156 (77%) of a total of 1494 students returned their forms and 883 students (59%) agreed to participate in the study. Only adolescents for whom full consent (their own and their parents) was obtained were enrolled in the study.

#### Procedure

##### Procedure of the effectiveness study

To assess the outcome measures for the effectiveness study, a variety of measurements/instruments were used: questionnaires, body measurements, shuttle-run tests, and pedometers. To discuss in which lessons these measurements would be conducted, appointments were made with school representatives. The computer questionnaires were completed under supervision of a researcher during a class hour. The researcher explained the procedure for the completion of the questionnaire at the beginning of the lesson and was available to answer questions. Non-participating students were instructed by the teacher to do something else. Pedometers and diary forms were handed out to five randomly chosen students of each class, who would wear them for one week (after completion of the questionnaire). After one week a researcher would collect the pedometers and forms. Weight, height, and waist circumference were measured at school one week after completion of the questionnaire by trained researchers and assistants. Students were individually measured to ensure privacy. Fitness was measured with a shuttle-run test performed by the physical activity teacher according to our protocol. Length of the circuit was measured to correct for gyms that are shorter than 20 meters. The measurement scheme is shown in Figure 2. Height and weight, waist circumference, and aerobic fitness were measured at baseline and 2-year follow-up. The behaviours and behavioural determinants were measured at baseline and at both follow-up points.

##### Procedure of the process evaluation

A process evaluation among students and teachers will provide information on the appreciation of the intervention, on the quality of the implementation of the intervention in schools, and usability in teaching practice. The students' process evaluation questionnaire was administered as part of the final module of the intervention. The teachers were asked to complete a logbook, which was part of the teacher manual. In addition, a telephone interview was held with each teacher who worked with the intervention.

##### Intervention implementation

The intervention was implemented by the teachers. Teachers received a teacher manual including instructions on the implementation schedule, on the content and procedure of working with the website itself, and on trouble-shooting. In addition, all teachers were phoned at the start of the intervention period to see if any problems that might hinder the implementation could occur. Practical problems were then solved. The implementation instructions were to work through the 8 modules in 8 lessons in sessions of about 15 minutes. In the last session the students had to go to 'Your advice' and read the most important advice again. Within a 10-week intervention period the teachers had to implement this 8-weeks program, preferably one session each week in 8 subsequent weeks, but with a 2-week margin. The teachers received passwords for all students to enter the program. In two of the intervention schools the researchers assisted with the implementation of the program because these schools had a lack of school staff during the implementation period.

#### Measurements

##### Body measurements

The BMI, calculated as weight (in kilograms) divided by height squared (in meters), and waist circumference were measured by trained research assistants, following a protocol. Body height was measured twice without shoes using a Seca 225 mobile height rod with an accuracy of 0.1 cm. A calibrated electronic digital floor scale (SECA 888 class III) was used to measure, with an accuracy of 0.2 kilogram, the body weight of the students, who were wearing sports clothes or their underwear and a T-shirt. Waist circumference was measured twice using flexible bands of Seca, with an accuracy of 0.1 cm. In case of a difference of more than 1.0 cm between these two measurements, the waist circumference was measured twice again.

##### Questionnaires

Electronic, self-administered questionnaires were used to assess self-reported behaviour, behavioural determinants, and potential behavioural determinants regarding prevention of getting fat. Completion of the questionnaire took about 30 to 60 minutes.

#####         Behaviour

Physical activity and sedentary behaviour were assessed using an adapted version of the Flemish validated questionnaire [29]. This questionnaire assesses physical activity at school and during leisure time (e.g. sports), active transportation to school, television viewing, and computer use during leisure time in the past seven days by asking about the frequency and duration of the activities. The questionnaire contains 31 questions. Test-retest reliability coefficients for the physical activity sub-behaviours vary between 0.68 and 1.00.

Dietary intake was assessed using a food frequency questionnaire assessing the frequency and quantity of a variety of foods (snacks, soft drinks, fruit and vegetable, fibre-rich products) eaten in the past week and a self-administered 24-hour recall of the specific food items. Adapted versions of validated questionnaires were used for snacks intake [30] and for fruit and vegetables intake [31]. The dietary questionnaire contains 39 questions.

#####         Determinants of the behaviours

Attitude, subjective norm, perceived behavioural control, and intention were assessed with respect to changing the desired behaviour (e.g. increase physical activity). All the questions on the TPB variables, and availability and accessibility, were measured on a 5-point bipolar scale. Attitude was measured with two items regarding the beliefs good/bad, and helpful/not helpful to prevent getting fat (e.g. For me drinking less sugar-sweetened beverages is very bad – very good) and subjective norm with one (e.g. Do you think your parents want you to eat more vegetables? Certainly yes-certainly not). Perceived behavioural control was measured with two items covering the dimensions easy/difficult (e.g. Do you think it is very difficult or very easy to eat more vegetables?), and the likelihood of succeeding (e.g. Do you think you will succeed in eating more fruit if you want to? Certainly yes-certainly not). Intention to change the behaviour was assessed with one item (e.g. Do you intend to exercise more in the upcoming year? Certainly yes-certainly not). Self-rated intake was assessed with one item (e.g. Do you think you drink a lot or very few sugar-sweetened beverages?). In addition, availability and accessibility of food and opportunity to exercise at home and at school were assessed with two items (e.g. Are there sugar-sweetened beverages available at home? and Are you allowed to drink as many sugar-sweetened beverages as you like at home? Always-never). These items were assessed as potential moderators of intervention effects. Conscientiousness, one of the 'big five' personality traits, was measured using the short Dutch version of Big Five personality trait [32], with test-retest reliability coefficients of about 0.7. In total there were 60 behavioural determinant questions.

#####         Prevention of getting fat

Questions about the concept of getting fat were included. Questions were measured on a five-point bipolar scale. Perception of a student's weight was measured with two items: Do you consider yourself as being too lean – too fat? and Do you consider yourself as being leaner – fatter than others of your age and gender? Attitude was assessed with two items: Do you consider preventing getting fat as good – bad? and Do you consider preventing getting fat as important – unimportant? Subjective norm was assessed by one item: Do you think your parents want you to prevent getting fat? Certainly yes-certainly not. Perceived behavioural control was assessed with two items: Do you think it is very difficult or very easy to prevent getting fat? and Do you think you will succeed in preventing getting fat? Certainly yes-certainly not. Intention was measured with one item: Do you intend to prevent getting fat in the coming two years? Certainly yes-certainly not. Perception of the risk of getting fat was measured with two items: Do you think you have a very high – very low chance getting fat in the coming two years? and Do you think you have a much larger – much lower chance of getting fat in the upcoming two years compared to others of your age and gender? Stage of change related to prevention of weight gain was assessed using a staging algorithm based on the PAPM: what statement fits you best? I've never thought of preventing getting fat – I already take action to prevent getting fat. In addition, we asked whether the students felt in control over the prevention of getting fat with two items: Do you think you can do something yourself not to get fat? Certainly yes-certainly not, and State five things you can do to prevent getting fat. In addition, we assessed overweight/obesity in the students' social environment: Is someone in your family fat? No, yes one, yes more than one; Do you have friends who are too fat? No, yes one, yes more than one. In total there were 15 questions about the prevention of getting fat.

#####         Demographics

Demographic questions included gender, age, educational level, country of birth, parents' country of birth, and postal code.

##### Pedometers

Physical activity level was measured with pedometers (Digiwalker SW200, NewLifelstyles) in a random sub-sample of five students per class. Pedometers have shown to be a valid and reliable tool for assessment of physical activity [33,34]. Students wore the pedometers for seven consecutive days on their belt or waistband on one side of the body. A diary was used to register the daily step count and periods/activities performed without wearing the pedometer.

##### Shuttle-run test

Aerobic fitness was measured with a shuttle-run test administered during physical exercise lessons. The shuttle-run test is a validated test which measures aerobic capacity by running back and forth for 20 meters, with an initial running pace of 8.0 km/h and a progressive 0.5 km/min increase of the running speed indicated by a sound [35]. The maximal performance is reached when the student does not cross the 20-m line at the moment of the beep for two consecutive 20-m distances. The number of minutes ran were counted with a precision of 0.5 minutes. Because not all school gyms are 20 meter in length, the area where the shuttle-run test takes place was measured to correct for differences between schools. The outcome was registered by the teacher and copied by the researchers.

##### Process measures

The students' questionnaire consisted of 24 questions using a 5-point bipolar scale. Topics covered for each module were: appreciation, personal relevance, and usefulness of the information. General questions were asked about: reading the information, printing the advice, reading the printouts afterwards, discussing the advice with parents and peers, using the advice in practice, considering the topic weight gain more often after finishing the program, the length of the modules, comprehensiveness of the information, finding the information interesting, learning new things, appearance, usability, and importance of the subject of weight gain prevention. In addition, we asked students to rate the program from 1 to 10 and to state the two most and the two least interesting topics.

A detailed description of the implementation of the intervention in each class was constructed from the logbook, teachers' interviews, and server information (how the modules were used, whether all the modules were used, what was the time needed to implement each module). In addition, the teachers' interviews were used to explore the following topics: opinions about the barriers and facilitators for implementation (implementation in school setting and daily practice, responses of the students), teacher manual (is it used, is it clear), the teachers' opinion about the topic and the content of the CTI, and the participation of student in the study (to have qualitative views on the participation rates).

#### Statistical analysis

Multilevel regression analysis will be used to test for post-test group differences on the primary outcome measures. This technique adjusts for the dependency between observations of students from the same school [36].

## Discussion

In this paper we outlined the steps in the planned development of the FATaintPHAT intervention and the protocol for the effectiveness and process evaluation. This study is one of the first CTIs to address seven EBRBs, and one of the first to evaluate a stand-alone CTI for adolescents. The intervention is an extensive CTI that targets the important EBRBs at both sides of the energy balance, i.e. eating and physical activity, in adolescents. Targeting seven important behaviours is not often applied in intervention research, but might be important because of the complicated functioning of the EBRBs together and because of limited positive effects reported in studies where only a few behaviours were targeted [16]. Many studies have shown that computer tailoring is a promising technique for changing health behaviours [11]. However, we do not know if this technique is useful for the prevention of excessive weight gain in adolescents. Our study enables to study the effectiveness of computer tailoring among adolescents.

The strengths of this study design are the size of the study (20 school), the random controlled design, the short-term (4 months) and long-term follow-up (2 years), and the inclusion of objective measures of BMI and physical activity. Limitations of the study are that not all outcome measures have validated instruments available, and that some outcome measures are based on self-reports. In addition, because of including multiple-risk behaviours in the present study, we had limited space in the questionnaire to assess behavioural determinants in detail.

The results of the study will contribute to the body of evidence on computer tailoring and particularly to the effectiveness on weight gain, EBRBs, and the application in adolescents. In addition, if the intervention proves to be effective we will have a useful application that can readily be implemented in secondary schools, where there is a need for educational programs on weight maintenance.

## Competing interests

The author(s) declare that they have no competing interests.

## Authors' contributions

NE, AO, and JB contributed to the design of the intervention and the study and to the drafting of the manuscript. PLJ was involved in the design of the study and in drafting the manuscript. All authors read and approved the final manuscript.

## Pre-publication history

The pre-publication history for this paper can be accessed here:


